# Abnormally high plasma concentrations of M-4, the active metabolite of edoxaban, at the onset of acute kidney injury in a patient receiving rifampin and clarithromycin: a case report

**DOI:** 10.1186/s40780-024-00390-6

**Published:** 2024-10-28

**Authors:** Junichi Nakagawa, Keinosuke Ishido, Norihisa Kimura, Hayato Nagase, Yusuke Wakasa, Satoshi Yokoyama, Kayo Ueno, Kenichi Hakamada, Takenori Niioka

**Affiliations:** 1 Department of Pharmacy, Hirosaki University Hospital, 53 Hon-Cho, Hirosaki, Aomori 036-8563 Japan; 2 Department of Gastroenterological Surgery, Hirosaki University Graduate School of Medicine, 5 Zaifu-cho, Hirosaki, Aomori 036-8562 Japan; 3 Department of Pharmaceutical Science, Hirosaki University Graduate School of Medicine, 53 Hon-cho, Hirosaki, Aomori 036-8563 Japan

**Keywords:** Edoxaban, M-4, Rifampin, Clarithromycin, Drug-drug interaction, Acute kidney injury, Pharmacokinetics

## Abstract

**Background:**

Edoxaban, the only factor Xa inhibitor with active metabolites, is metabolized by carboxylesterase-1 to its main active metabolite, M-4, which is a substrate of organic anion transporting polypeptide 1B1 (OATP1B1) and is excreted in bile and urine. Because the area under the plasma concentration–time curve ratio of M-4 to parent compound is typically less than 10% in healthy subjects, M-4 is generally considered to exhibit negligible antithrombotic activity in patients treated with edoxaban. However, we identified a case in which drug interactions and kidney impairment led to a substantive increase in plasma M-4 concentrations.

**Case presentation:**

This case report involved a 68-year-old man with pancreatic cancer who was orally administered edoxaban tablets for prevention of thrombus formation in non-valvular atrial fibrillation, in addition to rifampin and clarithromycin (CAM) for treatment of mycobacterium avium complex lung disease. These medications were temporarily discontinued for a pancreaticoduodenectomy but were resumed 8 days post-surgery (POD8). On POD9, the patient developed acute kidney injury, and the trough concentrations of edoxaban and M-4 were 131.1 ng/mL and 115.8 ng/mL, respectively (M-4 ratio: 88.3%). On POD11, the M-4 trough concentration and M-4 ratio increased to 216.2 ng/mL and 186.2%, respectively. The plasma concentration of coproporphyrin-I, an endogenous biomarker of OATP1B1 activity, increased during this period.

**Conclusions:**

This case suggests that in patients with impaired renal function taking edoxaban, co-administration of carboxylesterase-1 inducers such as rifampin and/or OATP1B1 inhibitors such as rifampin or clarithromycin may increase plasma concentrations of M-4 to clinically non-negligible levels.

## Introduction

Edoxaban, a direct oral anticoagulant, is a reversible inhibitor of factor Xa, and the anti-factor Xa activity of edoxaban exhibits a close direct linear relationship with its plasma concentration [1]. The bioavailability of edoxaban is approximately 62%, and approximately 50% of the absorbed drug is excreted in its unchanged form through the kidneys, and a portion of the other 50% is metabolized by the liver and excreted in bile [1]. Edoxaban is metabolized by carboxylesterase-1 (CES1) to its main active metabolite, M-4. Edoxaban is also a substrate of P-glycoprotein (P-gp; gene code *ABCB1*), and M-4 is a substrate of organic anion transporting polypeptide 1B1 (OATP1B1; *SLCO1B1*) [2].

Because the area under the plasma concentration–time curve (AUC) ratio of M-4 to parent compound has been found to be less than 10% in healthy subjects [1], this active metabolite is generally considered to exhibit little antithrombotic activity in patients treated with edoxaban. However, in a previous study, we found that in patients with non-valvular atrial fibrillation (NVAF) who were administered edoxaban, the M-4 ratio at the steady state trough varied widely among individuals, with the level in some patients reaching almost 80% [3]. In addition, in cases of concomitant use of an OATP1B1 inhibitor and edoxaban, the AUC of M-4 and the M-4-to-edoxaban AUC ratio increased significantly; under these conditions, previous studies have provided evidence consistent with a substantial antithrombotic effect of M-4 [4, 5]. M-4 has an anti-factor Xa activity with a potency similar to that of the parent compound (IC_50_: 1.8 nM [0.94 ng/mL] vs. 3 nM [1.64 ng/mL], respectively) [1], but the albumin binding rates of M-4 and edoxaban are different (80% vs. 55%, respectively) [2]. Therefore, further investigation is needed regarding the pharmacokinetics (PK)-pharmacodynamics of M-4 in clinical practice.

As previously mentioned, multiple drug-metabolizing enzymes and transporters are involved in the PK of edoxaban and M-4. Therefore, close attention should be paid to potential pharmacokinetic interactions in drug therapies involving these inhibitors or inducers. The incidence of lung disease caused by infection with mycobacterium avium complex (MAC) continues to increase, and patients infected with MAC are generally treated with a three-drug chemotherapy regimen of ethambutol, rifampin (RFP), and the macrolide antibiotic clarithromycin (CAM). CAM is an inhibitor of cytochrome P450 (CYP)3A, P-gp, and OATP1B1, and it has been reported to increase the AUC of edoxaban [6]. In contrast, RFP is an inducer of the drug-metabolizing enzymes, such as CYP3A and CES1, and an inducer/inhibitor of the drug transporters, and it has been reported to decrease the AUC of edoxaban and to increase the AUC of M-4 [5].

While the opposing effects of CAM and RFP on the activities of CYP3A and P-gp have been well established, the effects of these drugs on OATP1B1 activity remain controversial. While CAM and single-dose RFP are known to inhibit OATP1B1, it has been suggested that chronic RFP administration may induce OATP1B1 [7]. An additional factor complicating pharmacokinetic analyses of edoxaban is the reported down-regulation of various drug-metabolizing enzymes and transporters by inflammatory cytokines, including interleukin (IL)-6 [8].

We experienced a patient with MAC lung disease taking edoxaban and a chemotherapy combination of CAM, ethambutol, and RFP who had acute kidney injury (AKI) with an inflammatory response after pancreatoduodenectomy. We report here kinetic analyses of the plasma concentrations of edoxaban and M-4 in this case.

## Case presentation

**Fig. 1:**
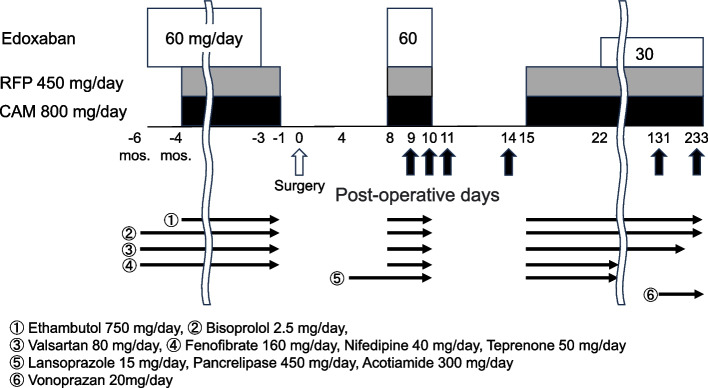
Schedule of drug administration and blood collection. Vertical black arrows indicate blood collection for plasma drug concentration measurements. CAM, clarithromycin; RFP, rifampin

The patient was a 68-year-old man weighing 55.5 kg. The patient had pancreatic cancer and was admitted to Hirosaki University hospital to undergo a pancreatoduodenectomy. Written informed consent was obtained from this patient for the measurement of each biomarker and polymorphisms of pharmacokinetics-related genes. The patient had been taking edoxaban tablets (Lixiana®), 60 mg once per day after breakfast for 6 months prior to admission to prevent recurrence of thrombus formation due to NVAF. The dose of edoxaban was determined based on the patient's weight at the start of edoxaban administration (> 60 kg), and the dose remained constant throughout the treatment. In addition, two months after the start of edoxaban therapy, the patient began a three-drug regimen for the treatment of MAC lung disease. This regimen involved taking CAM 400 mg twice per day after breakfast and supper, RFP 450 mg once per day after breakfast, and ethambutol 750 mg once per day. Other concomitant medications administered to this patient are shown in Fig. 1.

As shown in Fig. 1, edoxaban was discontinued three days before surgery, and all other drugs were discontinued on the day before surgery. All other medications, including edoxaban, RFP, and CAM, were resumed on POD8.

From POD9 through POD11, the patient developed AKI with dehydration due to insufficient water intake. As shown in Table 1, over those three days, the patient’s serum creatinine (S-CRE) level increased from 1.20 to 2.32 mg/dL. On the other hand, no abnormal liver function test values such as aspartate transaminase, alanine transaminase, or serum total bilirubin were observed after POD6. All oral medications were discontinued after administration on POD10 due to delayed gastric emptying. Although the patient developed a small area of subcutaneous haemorrhage in the left leg on POD10, the patient did not develop major bleeding during the observation period up to POD233. On POD15, the treatment regimen involving CAM, RFP, and ethambutol was resumed at the same doses as before discontinuation. Treatment with edoxaban tablets was resumed at the reduced dose of 30 mg/day on POD22, and the patient was discharged on POD22.

**Table 1:** Plasma drug concentrations and laboratory test values

	Post-operative day						
	6	9	10	11	14	131	233
Time from last administration of edoxaban tablet to blood collection (h)	-	24	8	24	96	26	2
Plasma concentration of edoxaban (ng/mL)	-	131.1	796.2	116.1	< 5.0	< 5.0	413.2
Plasma concentration of M-4 (ng/mL)	-	115.8	878.5	216.2	2.1	< 2.0	189.7
M-4 ratio (%)	-	88.3	110.3	186.2	-	-	45.9
Plasma concentration of coproporphyrin-I (ng/mL)	2.4	4.6	7.4	6.0	0.8	0.6	1.0
Plasma concentration of valsartan (µg/mL)	2.4	5.7	9.4	10.4	1.4	1.0	-^b^
Plasma concentration of IL-6 (pg/mL)	18.0	20.1	76.0	23.6	7.2	5.2	< 3.1
Serum C-reactive protein (mg/L)	66.4	50.5	64.2	147.1	17.4	6.0	14.0
Serum creatinine (mg/dL)	0.88	1.20	2.21	2.32	0.96	0.89	0.89
Creatinine clearance^a^ (mL/min)	62.5	46.3	25.1	23.9	57.8	59.0	62.4
Body weight (kg)	55.5	-	-	-	-	52.5	58.0

The plasma concentrations of edoxaban, M-4, CP-I and valsartan were measured by UPLC-MSMS, and those of IL-6 by Human IL-6 Quantikine ELISA Kit (R&D Systems, Inc.)

*CP-I* coproporphyrin-I, *IL-6* interleukin-6

^a^Creatinine clearance estimated by the Cockcroft-Gault formula

^b^Valsartan was discontinued between POD131 and 233

The plasma concentrations of edoxaban and M-4 and the plasma concentration ratios of M-4 to edoxaban (M-4 ratio) after surgery are shown in Fig. 1 and Table 1. On POD9, the trough concentrations of edoxaban and M-4 (C_0h-Edo_ and C_0h-M-4_) were 131.1 and 115.8 ng/mL, respectively, and the M-4 ratio was 88.3%. On POD10, the plasma concentrations of edoxaban and M-4 at 8 h after administration of an edoxaban tablet (C_8h-Edo_ and C_8h-M-4_) were 796.2 and 878.5 ng/mL, respectively, and the M-4 ratio was 110.3%. On POD11, C_0h-Edo_ was 116.1 ng/mL, but C_0h-M-4_ and the M-4 ratio increased to 216.2 ng/mL and 186.2%, respectively. During an outpatient follow-up on POD131, C_0h-Edo_ and C_0h-M-4_ were determined to be below the lower limits of quantification; interviews with the patient on POD131 confirmed that edoxaban was taken the previous morning. On POD233, the plasma concentrations of edoxaban and M-4 at 2 h after administration (C_2h-Edo_ and C_2h-M-4_) were 413.2 and 189.7 ng/mL, respectively, and the M-4 ratio was 45.9%.

Coproporphyrin-I (CP-I), valsartan, and IL-6 were measured in the same blood samples used to measure plasma concentrations of edoxaban and M-4. Plasma concentrations of CP-I at trough on POD6, 9, 11, and 131 were 2.4, 4.6, 6.0, and 0.6 ng/mL, respectively. Plasma concentrations of valsartan at the same trough were 2.4, 5.7, 10.4, and 1.0 µg/mL, respectively. Plasma concentrations of IL-6 on POD6, 9, 10, and 11 were 18.0, 20.1, 76.0, and 23.6 pg/mL, respectively.

The drug metabolizing enzyme and transporter gene polymorphisms of this patient were determined with real-time PCR using TaqMan probes. These polymorphisms were found to be CYP3A5*1/*3; *SLCO1B1* 521 T/C and 388G/G; and *ABCB1* 1236 T/T, 2677G/T, and 3435C/C. The patient's *SLCO1B1* haplotype was classified as *1b (c.388G – c.521 T)/*15 (c.388G – c.521C).

## Discussion

One point of interest in this case is that the patient’s M-4 ratio was markedly higher than that observed in typical patients. Specifically, the patient’s M-4 ratio reached more than twice the upper limit observed in our previous study (186.2%) [3]. Therefore, according to the findings of this case report, clinicians should recognize that high plasma concentrations of M-4 can be achieved and that it is possible for the plasma concentration of M-4 to be equal to or higher than that of edoxaban. In our situation, the measurement of edoxaban and M-4 plasma concentrations on POD11 led to the temporary discontinuation and subsequent reinitiation of edoxaban at a reduced dose, which likely contributed to the prevention of a major bleeding event.

A second point of interest is the nearly twofold increase in the M-4 ratio during the period when S-CRE increased from 1.20 mg/dL (POD9) to 2.21 mg/dL (POD11). The rate of increase in the plasma concentrations of edoxaban and M-4 due to renal impairment has been reported to be similar [9]. Therefore, the marked increase in the M-4 ratio during this period was likely due to drug-drug interactions of M-4 with the OATP1B1 inhibitors CAM and RFP. Since edoxaban is not an OATP1B1 substrate [2], an increase in the M-4 ratio would be expected with concomitant administration of OATP1B1 inhibitors. Simulations based on a PBPK model have shown that the anticoagulation effect associated with increased plasma concentrations of M-4 cannot be ignored when OATP1B1 is inhibited or downregulated, especially when combined with renal impairment [4]. We also found that plasma concentrations of valsartan and CP-I, which are substrates of OATP1B1, were elevated during this period. Because only 13% of unchanged valsartan is excreted in the urine and the PK of valsartan are not affected by renal function [10], we conclude that the activity of OATP1B1 was reduced during this period. Furthermore, since polymorphisms in the gene encoding OATP1B1 affect the PK of M-4 [3], we considered that these polymorphisms could also influence the interactions of edoxaban with CAM and RFP. The patient in this case had the SLCO1B1*1b/*15 haplotype, indicating that OATP1B1 activity was functional; thus, it is possible that the increase in plasma M-4 concentrations was mediated by inhibition of OATP1B1 by CAM and RFP. In addition to drug-drug interactions and AKI, the presence of inflammatory cytokines may have also been involved in the marked increase in M-4 plasma concentrations. These cytokines likely led to decreased activity of drug-metabolizing enzymes and drug transporters, further increasing the plasma concentrations of edoxaban, CAM, and RFP and exacerbating the drug-drug interactions.

A third point of interest is the low C_0h_ and high near peak concentrations (C_2h_) of edoxaban (< 5.0 and 413.2 ng/mL) and M-4 (< 2.0 and 189.7 ng/mL) at the outpatient follow-up visits (POD131 and POD233) during which improved renal function and inflammatory response were observed (S-CRE, 0.89 and 0.89 mg/dL; serum C-reactive protein, 6.0 and 14.0 mg/L). It is likely that in the present case, excretion of edoxaban, M-4, CAM [11], and RFP [12] increased with recovery of renal function, and the effects of kinetic interactions among these drugs via OATP1B1 were reduced, resulting in lower C_0h-Edo_ and C_0h-M-4_. On the other hand, the C_2h-Edo_ and C_2h-M-4_ on POD233 in this case were higher than the maximum plasma concentration (C_max_) of edoxaban and M-4 in healthy subjects administered edoxaban at 60 mg/day [1]. A possible reason for the high C_2h-Edo_ and C_2h-M-4_ could be the inhibition of P-gp in the small intestine by CAM, but since RFP is known to induce P-gp, the mechanisms underlying these drug interactions require further investigation.

A limitation of this study is that we did not evaluate whether the plasma concentrations of edoxaban and M-4 observed during outpatient follow-up visits fell within the ranges that would be predicted with previously reported PK model simulations [4]. Such evaluations would require planned blood sampling and the calculation of PK parameters based on plasma concentration measurements. Additional studies would provide further insights into the mechanisms underlying the drug-drug interactions that we observed in this case.

In summary, we were able to confirm the existence of a case with much higher M-4 plasma concentrations and M-4 ratios than previously reported. In patients with impaired renal function, co-administration of OATP1B1 inhibitors may increase plasma concentrations of M-4 with factor Xa inhibition activity to clinically non-negligible levels.

## Data Availability

The datasets used and/or analysed during the current study are available from the corresponding author on reasonable request.

## Abbreviations

AKI: Acute kidney injury
AUC: Area under the plasma concentration–time curve
CAM: Clarithromycin
CES1: Carboxylesterase-1
CP-I: Coproporphyrin-I
C_max_: Maximum plasma concentration
CYP: Cytochrome P450
IL-6: Interleukin-6
MAC: Mycobacterium avium complex
M-4 ratio: Plasma concentration ratio of M-4 to edoxaban
NVAF: Non-valvular atrial fibrillation
OATP1B1: Organic anion transporting polypeptide 1B1
P-gp: P-glycoprotein
PK: Pharmacokinetics
POD: Post-operative day
RFP: Rifampin
S-CRE: Serum creatinine
